# An Analytic Compact Model for P-Type Quasi-Ballistic/Ballistic Nanowire GAA MOSFETs Incorporating DIBL Effect

**DOI:** 10.3390/nano15221734

**Published:** 2025-11-17

**Authors:** He Cheng, Zhijia Yang, Chao Zhang, Zhipeng Zhang

**Affiliations:** 1 State Key Laboratory of Robotics and Intelligent Systems, Shenyang Institute of Automation, Chinese Academy of Sciences, Shenyang 110016, China; chenghe@sia.cn (H.C.); yang@sia.cn (Z.Y.); zhangchao@sia.cn (C.Z.); 2 Shenyang Institute of Automation, Chinese Academy of Sciences, Shenyang 110016, China

**Keywords:** quasi-ballistic transport, compact model, Verilog-A, p-type GAA MOSFET, DIBL effect

## Abstract

We present an analytic compact model for p-type cylindrical gate-all-around (GAA) MOSFETs in the quasi-ballistic/ballistic regime, incorporating drain-induced barrier lowering (DIBL). To describe the potential profile, an undetermined parameter is used to represent the channel potential, which is derived from the Laplace equation in the subthreshold region and from Gauss’s law combined with quantum statistics in the inversion region. A smoothing function is applied to this parameter to ensure a continuous source—drain current across all operating regions. The current model is based on the Landauer approach and captures both quasi-ballistic/ballistic transport and quantum-confinement effects. It is validated against non-equilibrium Green’s function (NEGF) simulation results and implemented in Verilog-A for SPICE circuit-level simulation of a CMOS inverter, demonstrating its applicability for nanoscale design.

## 1. Introduction

The continued scaling of MOSFET dimensions below 10 nm has made strong gate control essential to reduce short-channel effects (SCEs), such as threshold voltage roll-off and drain-induced barrier lowering (DIBL) [1,2,3,4]. At the same time, the silicon body thickness has also been scaled below 10 nm, as predicted by the International Roadmap for Devices and Systems (IRDS) [5]. Among various advanced device structures, gate-all-around (GAA) MOSFETs are promising for future CMOS technologies due to their better electrostatic control and improved SCE suppression. To further increase integration density in a limited footprint, multi-stacked silicon nanosheet GAA structures have been proposed to widen the channel effectively [6,7,8,9]. Although nanosheet GAA FETs has been commercially adopted, nanowire GAA remains valuable as an analytically tractable platform for sub-10 nm studies of ballistic transport and quantum confinement. Its cylindrical geometry enables closed-form treatments of SCE, supporting compact model development. The resulting framework is readily extendable to nanosheet GAA FETs or complementary FETs (CFETs) via appropriate subband/density-of-states (DOS) formulations and geometry-aware electrostatics. Cylindrical GAA MOSFETs have been widely considered as successors to FinFETs at advanced technology nodes, with commercial adoption beginning at the 3 nm node, owing to their excellent subthreshold swing (SS) and high $I_{\mathrm{ON}}/I_{\mathrm{OFF}}$ ratio [7,10,11]. GAA MOSFETs also show potential in specialized applications such as radiation-hardened circuits and gas sensors [12,13]. Since the channel lengths considered (<10 nm) are on the order of the hole mean free path in silicon [14], transport lies in the quasi-ballistic to ballistic regime, providing a practical guideline indicating that channel lengths comparable to the mean free path are the range where ballistic effects become relevant. As the silicon thickness and channel length continue to shrink, quantum-confinement and ballistic transport effects become more important and cannot be ignored. Phonon processes in low-dimensional structures play a crucial role in carrier transport and scattering mechanisms. Relevant studies include [15], along with experimental and theoretical investigations, such as [16]. These works provide important context for understanding phonon interactions in nanostructured devices. Thus, accurate models must include both quantum and ballistic effects.

Carrier transport in both n-type and p-type GAA MOSFETs under quantum and ballistic conditions can be accurately described using advanced numerical methods, such as the non-equilibrium Green’s function (NEGF) method, ab initio simulations, and the k·p method [9,13,17,18,19,20]. However, the high computational cost of these methods makes them impractical for circuit-level simulations. To solve this problem, several analytical compact models have been developed for short-channel ballistic GAA MOSFETs [21,22,23,24,25]. The models describing the I−V behavior under ballistic transport and include effects such as thermionic emission and source-to-drain tunneling (SDT) are reported [26,27]. Nevertheless, most existing models focus only on n-type devices. Due to the anisotropic valence band, p-type devices exhibit stronger orientation dependence, leading to large variations in hole effective mass and mobility. Therefore, a compact model for short-channel p-type GAA MOSFETs that includes the DIBL effect and works across all operating regions remains lacking and is critically needed.

This paper presents an analytic compact model for p-type cylindrical GAA MOSFETs that includes quasi-ballistic/ballistic transport, quantum confinement, and the DIBL effects. The model is validated by technology computer-aided design (TCAD) simulations and implemented in Verilog-A. The remainder of this paper is organized as follows. Section 2 describes the device structure, defines key parameters, and formulates the quasi-ballistic/ballistic current model for p-type devices. Section 3 details the modeling of an unknown parameter in both subthreshold and inversion regions, along with the derivation of the lowest subband energy level across all operation modes. Section 4 presents the simulation results and discusses the accuracy and applicability of the proposed model. To the best of our knowledge, this is the first analytical model for p-type ballistic nanowire GAA MOSFETs that incorporates the DIBL effect and ballistic hole transport, validated in SPICE via Verilog-A.

## 2. Modeling Ballistic Current

### 2.1. Model Structure and Coordinates

**Figure 1:**
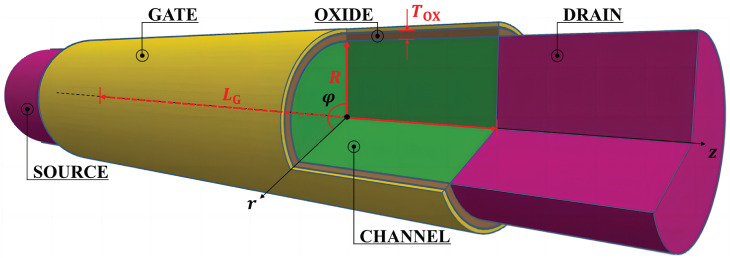
Schematic illustration of the nanowire GAA MOSFET structure. The channel length, nanowire radius, and gate oxide thickness are denoted by $L_{G}$, *R*, and $T_{\mathrm{OX}}$, respectively.

As shown in Figure 1, the device is modeled as a cylinder with a circular cross-section. The channel is made of intrinsic silicon, and the source/drain regions are heavily doped p-type silicon. The source and drain are treated as ideal contacts that inject carriers without backscattering. The channel is fully surrounded by a metal gate and SiO_2_ gate oxide, forming a GAA structure.

In the following analysis, cylindrical coordinates are used with *z* along the channel, *r* as the radial coordinate, and φ as the angular coordinate. With perfect cylindrical symmetry assumed, the electrostatic potential and subband energy have no dependence on the angular coordinate φ. Therefore we can neglect φ in the analysis.

### 2.2. Quasi-Ballistic Current Derivation

**Figure 2:**
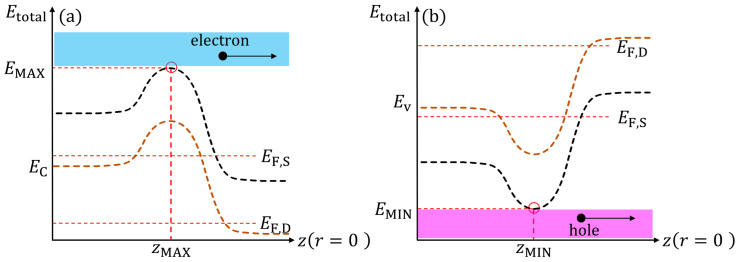
Simplified schematic of quasi-ballistic/ballistic transport along the *z*-axis for (**a**) electrons and (**b**) holes. $E_{C}$ and $E_{V}$ denote the conduction and valence band edges, shown as colored dashed lines. For electron transport in (**a**), positive gate and drain voltages are applied, and the subband energy maximum $E_{\mathrm{MAX}}$ defines the barrier top at $z_{\mathrm{MAX}}$. For hole transport in (**b**), negative gate and drain voltages are applied, and the subband energy minimum $E_{\mathrm{MIN}}$ occurs at $z_{\mathrm{MIN}}$. $E_{F,S}$ and $E_{F,D}$ represent the source and drain Fermi levels, respectively. This schematic highlights the contrast in energy barrier shapes between electron and hole transport.

Figure 2a,b illustrate the quasi-ballistic/ballistic transport mechanism along the channel for electrons and holes, respectively. In the quasi-ballistic/ballistic regime, carrier injection from the source is controlled by the potential barrier along the channel. For electrons, transport is determined by the maximum of the conduction subband profile, while for holes, it is determined by the minimum of the valence subband profile [28]. Thus, carrier transport in both cases is governed by the energy extremum of the corresponding subband profiles. The source—drain current primarily comes from carriers that have sufficient energy to overcome the barrier, a thermionic emission process described by the Landauer formalism. Accordingly, the expression for the quasi-ballistic/ballistic electron current in the device is given as follows [29,30]:

(1) $$\begin{array}{c} I_{\mathrm{DS}}=\frac{e}{\pi \hbar }\sum_{n_{\varphi },n_{r}}\sum_{n_{\nu }}\int_{0}^{\infty }dE_{z}\cdot g_{n_{\nu }}\left(1-R_{\mathrm{ref}}\right)\left[f_{e}\left(E_{F,S},E_{\mathrm{total}}\right)-f_{e}\left(E_{F,D},E_{\mathrm{total}}\right)\right], \end{array}$$

(2) $$E_{\mathrm{total}}=E_{\mathrm{MAX}}+E_{z},$$

(3) $$f_{e}\left(E_{F},E_{\mathrm{total}}\right)=\frac{1}{1+\exp\left(\frac{E_{\mathrm{total}}-E_{F}}{k_{B}T}\right)},$$

where *e*, *ℏ* and $k_{B}$ are the elementary charge, the reduced Planck constant and Boltzmann constant, respectively, $n_{\nu }$ denotes the specific valley index of the energy band, $g_{n_{\nu }}$ is the corresponding valley degeneracy, and $E_{\mathrm{MAX}}$ represents the maximum value of the subband energy level along the channel direction, defined by the angular and radial quantum numbers $n_{\varphi }$ and $n_{r}$, respectively, $R_{\mathrm{ref}}$ is the backscattering coefficient between the source and drain in quasi-ballistic transport, satisfying $0\leq R_{\mathrm{ref}}\leq 1$ [31,32]. The Fermi–Dirac distribution function for electrons, $f_{e}\left(E_{F},E_{\mathrm{total}}\right)$, is evaluated by the total carrier energy $E_{\mathrm{total}}$, which is defined with respect to the Fermi level $E_{F}$. The temperature *T* is given in Kelvin. On the other hand, based on Fermi–Dirac statistics describing the electron distribution in the channel as given by $1-f_{e}\left(E_{F},E_{\mathrm{total}}\right)$ [33].

In this study, only holes injected from the source to drain are considered to contribute to the quasi-ballistic/ballistic current in the p-type devices. The integration limits are reversed compared to electron transport because holes encounter a subband energy barrier at $E_{\mathrm{MIN}}$, which corresponds to the subband energy level in the valence band, as illustrated in Figure 2b. Since the total energy $E_{\mathrm{total}}$ of the hole lies below $E_{\mathrm{MIN}}$, the longitudinal kinetic energy, defined as $E_{z}=E_{\mathrm{total}}-E_{\mathrm{MIN}}$, is negative. As a result, the current integration is carried out over $E_{z}$ from −∞ to 0, unlike the electron case where $E_{z}$ is integrated from 0 to +∞. Therefore, the source-drain current can be expressed as follows:

(4) $$\begin{array}{c} I_{\mathrm{SD}}=\frac{ek_{B}T}{\pi \hbar }\sum_{n_{\varphi },n_{r}}\sum_{n_{\nu }}g_{n_{\nu }}\left(1-R_{\mathrm{ref}}\right)\ln\left[\frac{1+\exp\left(\frac{E_{n_{\varphi },n_{r}}-E_{F,S}}{k_{B}T}\right)}{1+\exp\left(\frac{E_{n_{\varphi },n_{r}}-E_{F,D}}{k_{B}T}\right)}\right]. \end{array}$$

(5) $$E_{F,D}=E_{F,S}-eV_{\mathrm{DS}},$$

where $V_{\mathrm{DS}}$ is defined as the source–drain voltage. While the general expressions above include all possible subbands characterized by the quantum numbers $n_{\varphi }$ and $n_{r}$, further simplifications will be introduced in the following sections.

## 3. Potential Profile Formulation

### 3.1. Subband Energy Profile and Definition of $\Delta U_{G}$

**Figure 3:**
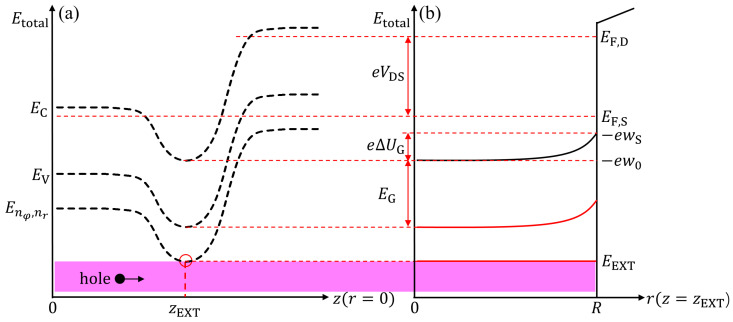
Rough sketches of the potential energy profiles along the channel and transverse directions, illustrating the carrier transport mechanisms in ballistic thermionic emission modes. (**a**) Energy level distribution along the *z*-direction at the channel center (r=0). (**b**) Schematic of the confinement potential energy along the *r*-direction at the subband energy minimum ($z=z_{\mathrm{EXT}}$) in the cross-section.

Figure 3a,b illustrate the conduction and valence band edge profiles ($E_{C}$ and $E_{V}$), along with the lower subband energy levels, corresponding to the potential distributions along the *z*-direction at r=0 and the *r*-direction at $z=z_{\mathrm{EXT}}$, respectively. As shown in Figure 3a, the hole subband energy level $E_{n_{\varphi },n_{r}}$ at $z=z_{\mathrm{EXT}}$ is defined as $E_{\mathrm{EXT}}$. In Figure 3b, the $E_{C}$ at the channel center (r=0) and the surface (r=R) are denoted as $-ew_{0}$ and $-ew_{S}$, which correspond to the electrostatic potentials $w_{0}$ and $w_{S}$, respectively. Thus, the potential difference between the center and surface of the channel, $w_{0}-w_{S}$, is characterized by an unknown parameter $\Delta U_{G}$. Then, $E_{n_{\varphi },n_{r}}$ is referenced to $E_{F,S}$, and can be obtained from the quantum confinement energy, which depends on the surface potential at $E_{V}$ along the channel. The subband energy levels of holes in the channel can thus be expressed as follows:

(6) $$E_{n_{\varphi },n_{r}}=-e\cdot w_{S}-E_{g}-E_{n_{\varphi },n_{r}}^{q},$$

(7) $$E_{n_{\varphi },n_{r}}^{q}=E_{n_{\varphi },n_{r}}^{q0}+e\cdot H_{n_{\varphi },n_{r}}\cdot \Delta U_{G},$$

(8) $$E_{n_{\varphi },n_{r}}^{q0}=\frac{\hbar^{2}}{2m_{\mathrm{xh}}^{r}}{\left(\frac{R_{n_{\varphi }}^{n_{r}}}{R}\right)}^{2},$$

where $E_{g}$ denotes the band gap between $E_{C}$ and $E_{V}$, so $E_{V}$ at surface is given by $-ew_{S}-E_{g}$, $E_{n_{\varphi },n_{r}}^{q}$ represents the confinement subband energy level of holes, referenced to $-ew_{S}-E_{g}$, while $E_{n_{\varphi },n_{r}}^{q0}$ denotes the unperturbed confinement energy level, $H_{n_{\varphi },n_{r}}$ is the perturbation matrix element, $m_{\mathrm{xh}}^{r}$ denotes the effective mass of confined holes along the radial direction, where the subscript x specifies the type of hole (heavy, light or split-off, denoted by h, l or s), and $R_{n_{\varphi }}^{n_{r}}$ is the $n_{r}$th zero of the first-kind Bessel function of order $n_{\varphi }$, as defined in [34]. However, the objective of analytic compact modeling is to derive an explicit analytical expression for $\Delta U_{G}$ as a function of the applied gate and drain biases. The following subsections will focus on formulating the expressions for $\Delta U_{G}$, addressing both the case with the DIBL effect and the long-channel case (without DIBL) in the subthreshold and inversion regions, respectively.

### 3.2. Analytical Solution for Channel Potential

To capture the DIBL effect in the subthreshold region, we derive an expression of $\Delta U_{G}$ as a function of the position along the *z*-direction. Based on previous studies, the electrostatic potential distribution in the channel can be obtained by solving the Laplace equation with appropriate boundary conditions. Hence, approximate analytic expressions for the electrostatic potential can be derived for both the radial and channel directions. As reported in [25], the electrostatic potential distribution is rewritten as follows:

(9) $$w\left(r,z\right)=\left[A\exp\left(\gamma \cdot z\right)+B\exp\left(-\gamma \cdot z\right)\right]\left(1-\frac{\gamma^{2}r^{2}}{4}\right)+V_{\mathrm{GS}}^{*},$$

(10) $$V_{\mathrm{GS}}^{*}=V_{\mathrm{GS}}-\varphi_{\mathrm{GC}}+w_{\mathrm{FB}},$$

where *A* and *B* are coefficients determined by boundary conditions at the drain and source ends of the channel, respectively, $V_{\mathrm{GS}}$ is the gate-source voltage, $\varphi_{\mathrm{GC}}$ represents the difference in work function between the gate and channel material, $w_{\mathrm{FB}}$ refers to $E_{C}$ under the flat band condition, and γ is a geometric scaling coefficient defined as follows:

(11) $$\gamma =\frac{2\cdot \beta }{R},$$

(12) $$\beta =\frac{1}{\sqrt{1+\frac{4\pi \epsilon_{\mathrm{CH}}}{C_{\mathrm{OX}}}}},$$

where $\epsilon_{\mathrm{CH}}$ is the dielectric constant of the channel, $C_{\mathrm{OX}}$ is the gate oxide capacitance per unit length. The boundary bias coefficients above are defined as follows:

(13) $$A=K\left\{\left(V_{\mathrm{bi}}-V_{\mathrm{GS}}^{*}\right)\left[1-\exp\left(-\gamma L_{G}\right)\right]+V_{\mathrm{DS}}\right\},$$

(14) $$B=K\left\{\left(V_{\mathrm{bi}}-V_{\mathrm{GS}}^{*}\right)\left[\exp\left(\gamma L_{G}\right)-1\right]-V_{\mathrm{DS}}\right\},$$

(15) $$K=\frac{8\beta^{2}}{\left(2-\beta^{2}\right)\left(4+\beta^{4}\right)}\cdot \frac{1}{\exp\left(\gamma L_{G}\right)-\exp\left(-\gamma L_{G}\right)},$$

where $V_{\mathrm{bi}}$ represents the junction built-in potential between the source and channel at equilibrium, which is determined by the electrostatic potential energy level of a subband at the source measured from $E_{F,S}/e$ [25]. Details on how $V_{\mathrm{bi}}$ is chosen are discussed in Section 4.

Next, by substituting Equation (11) into Equation (9), we obtain the expression of the electrostatic potential distribution in the channel as follows:

(16) $$w\left(r,z\right)=\left[A\exp\left(\gamma \cdot z\right)+B\exp\left(-\gamma \cdot z\right)\right]\left(1-\beta^{2}\frac{r^{2}}{R^{2}}\right)+V_{\mathrm{GS}}^{*}$$

By further simplifying, the radial dependence of the electrostatic potential is approximated by a quadratic function of *r*. Under this assumption, w(r,z) can be rewritten in a more compact form as follows:

(17) $$w\left(r,z\right)=w_{S}\left(z\right)+\left(1-\frac{r^{2}}{R^{2}}\right)\cdot \Delta U_{G}\left(z\right),$$

where $w_{S}\left(z\right)$ denotes the electrostatic potential of the channel surface along *z*-axis. According to the previous study [25,34], $w_{S}\left(z\right)$ can be written as:

(18) $$\begin{array}{c} w_{S}\left(z\right)=w\left(R,z\right)=V_{\mathrm{GS}}^{*}+V_{\mathrm{OX}}=V_{\mathrm{GS}}^{*}+\frac{4\pi \epsilon_{\mathrm{CH}}}{C_{\mathrm{OX}}}\Delta U_{G}\left(z\right), \end{array}$$

where $V_{\mathrm{OX}}$ is the potential difference across the oxide, $\Delta U_{G}\left(z\right)$ represents the *z*-component of potential variation due to DIBL, is expressed as:

(19) $$\Delta U_{G}\left(z\right)=\left[A\cdot \exp\left(\gamma \cdot z\right)+B\cdot \exp\left(-\gamma \cdot z\right)\right]\cdot \beta^{2}.$$

Furthermore, $z_{\mathrm{EXT}}$ is defined as the position along the channel where the subband energy level reaches its extremum as shown in Figure 3a, and can be expressed as:

(20) $$z_{\mathrm{EXT}}=\frac{1}{2\gamma }\ln\left(\frac{B}{A}\right).$$

The expression for $z_{\mathrm{EXT}}$ enables further analytical development of $\Delta U_{G}$ in the subthreshold region. In particular, substituting Equation (20) into Equation (19) gives $\Delta U_{G}\left(z\right)$ at $z_{\mathrm{EXT}}$ in terms of coefficients *A* and *B* (from Equations (13) and (14)):

(21) $$\Delta U_{G}^{\mathrm{DIBL}}\left(z_{\mathrm{EXT}}\right)=-2\sqrt{AB}.$$

**Figure 4:**
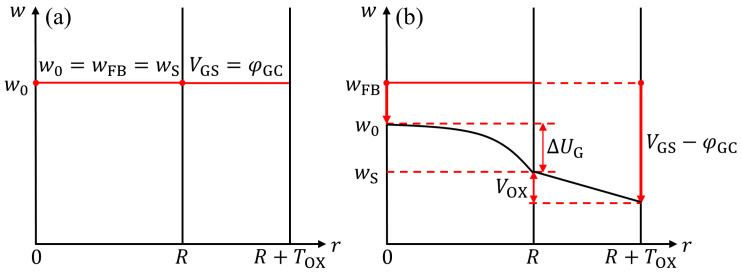
Schematic diagrams of electrostatic potential and potential distribution along *r*-component at $z=z_{\mathrm{EXT}}$. (**a**) Electrostatic potential distribution for flat-band condition. (**b**) Electrostatic potential distribution for $V_{\mathrm{GS}}<\varphi_{\mathrm{GC}}$ conditions.

Note that Equation (18) can also be derived from the relationships depicted in Figure 4a,b. In Figure 4a, the gate voltage $V_{\mathrm{GS}}$ equals the gate contact work function $\varphi_{\mathrm{GC}}$ under the flat-band condition. Both $w_{0}$ and $w_{S}$ are identical to the flat-band potential $w_{\mathrm{FB}}$. As $V_{\mathrm{GS}}$ decreases, as shown in Figure 4b, $V_{\mathrm{OX}}$ increases, leading to a corresponding reduction in $w_{S}$. Consequently, $w_{S}$ and $w_{0}$ diverge and their relationship can be approximated by a quadratic function of $\Delta U_{G}$ in the radial direction, as demonstrated in Equation (17).

### 3.3. $\Delta U_{G}$ for All Operation Regions

In the following analysis, the quasi-ballistic/ballistic current is assumed to consist of holes excited only to the lowest subband, characterized by quantum numbers $n_{\varphi }$ = 0 and $n_{r}$ = 1, across all operating regions. This assumption is based on the fact that, under typical bias conditions and device geometries, hole transport in p-type nanowire GAA MOSFETs is primarily governed by the lowest subband derived from the heavy-hole (HH) subband. Previous studies [26,35,36,37] have confirmed that the occupation of higher-order subbands is negligible for devices with R< 3 nm and $V_{\mathrm{GS}}<$ 0.4 V. Building on earlier modeling work for n-type devices without DIBL [34], where Gauss’s law was combined with quantum statistics to compute the carrier density at $z_{\mathrm{EXT}}$, we extend the approach to p-type devices. The corresponding $\Delta U_{G}$ at $z_{\mathrm{EXT}}$ is obtained numerically and expressed as:

(22) $$\begin{array}{cc} 4\pi \epsilon_{\mathrm{CH}}\Delta U_{G} & =\frac{e}{\pi \hbar }\sqrt{\frac{k_{B}Tm_{0}}{2}}\sum_{n_{\nu }}\\ \; & \;\times g_{n_{\nu }}\sqrt{\frac{m_{\mathrm{hh}}^{z}}{m_{0}}}\left[\left(1+R_{\mathrm{ref}}\right)f_{-\frac{1}{2}}\left(\frac{E_{0,1}-E_{F,S}}{k_{B}T}\right)\right.\\ \; & \;\;\;\;\left.+\left(1-R_{\mathrm{ref}}\right)f_{-\frac{1}{2}}\left(\frac{E_{0,1}-E_{F,D}}{k_{B}T}\right)\right], \end{array}$$

(23) $$F_{j}\left(a\right)=\int_{0}^{\infty }dy\frac{y^{j}}{1+\exp\left(y-a\right)},$$

where $m_{\mathrm{hh}}^{z}$ denotes the effective mass of the HH subband along the channel direction, $F_{j}$ represents the Fermi-Dirac integral function of order *j*. For sufficiently high drain bias ($|eV_{\mathrm{DS}}|\gg k_{B}T$), holes injected from the drain contribute negligibly to the thermionic current [34]. Accordingly, we neglect the second term on the right-hand side of Equation (22) in the analytic development and derive approximate closed-form expressions for $\Delta U_{G}$ in both the subthreshold and inversion regimes. To ensure continuity between the two regions, we then combine the two solutions into a unified expression $\Delta U_{G}^{\left(1\right)}$ using a smoothing function:

(24) $$\Delta U_{G}^{\left(1\right)}=\frac{C_{1}C_{2}}{2}\left\{\sqrt{1+\frac{4}{{C_{1}}^{2}C_{2}}\frac{1}{\alpha }\log\left[1+\exp\left(\alpha \cdot C_{3}\right)\right]}-1\right\},$$

(25) $$C_{1}=\frac{H_{0,1}}{V_{t}}+\frac{4\pi \epsilon_{\mathrm{CH}}}{C_{\mathrm{OX}}\cdot V_{t}},$$

(26) $$C_{2}=\frac{e^{2}k_{B}Tm_{\mathrm{hh}}^{z}{g_{\nu }}^{2}{\left(1+R_{\mathrm{ref}}\right)}^{2}}{8\pi^{4}\epsilon_{\mathrm{CH}}^{2}\hbar^{2}},$$

(27) $$C_{3}=-\frac{V_{\mathrm{GS}}^{*}}{V_{t}}-\frac{E_{0,1}^{q0}}{k_{B}T}-\frac{E_{g}}{k_{B}T},$$

where $V_{t}=k_{B}T/e$ is the thermal voltage, α is an empirical fitting parameter, set to 0.3 to achieve good agreement with NEGF simulation results for source—drain current characteristics in Section 4. Although it has no direct physical meaning, it ensures a smooth and differentiable transition between the subthreshold and inversion regions and is kept constant throughout the model to preserve consistency [36].

Finally, $\Delta U_{G}^{\mathrm{all}}$ at $z_{\mathrm{EXT}}$, valid across all operating regions, is formulated as follows:

(28) $$\Delta U_{G}^{\mathrm{all}}=\Delta U_{G}^{\mathrm{DIBL}}\left(z_{\mathrm{EXT}}\right)+\Delta U_{G}^{\left(1\right)}.$$

This expression represents the superposition of $\Delta U_{G}^{\mathrm{DIBL}}$ affected by DIBL in the subthreshold region and $\Delta U_{G}^{\left(1\right)}$ unaffected by DIBL in the inversion region, which means that the weak-inversion region lacks an explicit expression for $\Delta U_{G}^{\mathrm{all}}$. As a result, the lowest subband energy level for holes can be obtained by substituting Equation (28) into Equations (7) and (18).

## 4. Results and Discussion

For simplicity, the hole effective masses in the radial and channel directions are assumed equal ($m_{\mathrm{hh}}^{z}$ = $m_{\mathrm{hh}}^{r}$), neglecting the valence anisotropy and using 0.49$m_{0}$ as a representative value for the HH band in the given nanowire orientation [38]. Since the lowest subband comes from the HH band and dominates hole transport under normal conditions, the valence band is modeled as a single valley ($g_{n_{\nu }}$ = 1) [17]. A built-in potential of $V_{\mathrm{bi}}$ = −1.35 V, extracted from device simulations, is used for all cases to simplify calculations under different geometries and bias conditions [25]. In addition, we assume that $R_{\mathrm{ref}}$ is set as zero for all following calculations. Quantum reflection at the barrier and parasitic resistance at the source and drain are also ignored. This section compares the proposed model with TCAD simulations using six subbands calculations [38]. The TCAD simulations use a Poisson–NEGF (non-equilibrium Green’s function) framework under the effective-mass approximation (mode-space, Fermi statistics), solved self-consistently for a cylindrical Si/SiO_2_ nanowire. Geometry is swept over R=1.5,2.0,2.5 nm and L=7,9 nm, with 10-nm p-doped source/drain extensions and $T_{\mathrm{OX}}=0.5$ nm. The channel is undoped; the source/drain are uniformly p-doped to $1\times 10^{20}\;{\mathrm{cm}}^{-3}$. The gate work function is 4.8 eV (n-poly). Key NEGF controls include negf_ms, qcrit.negf, esize.negf, sp.smooth, num.band = 3, and eigen = 2. Full input decks, solver options, and scripts are available in the GitHub repository (see Data Availability Statement). NEGF simulations were performed with Silvaco ATLAS v5.22.1.R (DeckBuild). Circuit simulations used Cadence Spectre 15.1.0.284 with the BSIM-CMG 112.0.0 Verilog-A model. Analytical derivations were carried out in Wolfram Cloud (Mathematica), and data post-processing/plotting in R 4.5.1. In the following results, NEGF benchmarks confirm that this simplified HH-based treatment reproduces the main transport characteristics and orientation trends across the reported ranges of *L* and *R*.

The drain–source voltage is set to $V_{\mathrm{DS}}$ = −0.6 V, following the IRDS roadmap [5], and the source Fermi level $E_{F,S}$ is set to zero in the NEGF simulation. Moreover, in the quasi-ballistic/ballistic regime, orientation is captured via direction-specific heavy-hole masses: $m_{\mathrm{hh}}^{r}$ (radial, sets subbands) and $m_{\mathrm{hh}}^{z}$ (channel, sets injection velocity and 1D DOS), which together determine the Landauer current. Then, the source–drain current from Equation (4) can be rewritten as:

(29) $$I_{\mathrm{SD}}=\frac{ek_{B}T}{\pi \hbar }\cdot \ln\left[\frac{1+\exp\left(\frac{E_{0,1}\left(z_{\mathrm{EXT}}\right)}{k_{B}T}\right)}{1+\exp\left(\frac{E_{0,1}\left(z_{\mathrm{EXT}}\right)+eV_{\mathrm{DS}}}{k_{B}T}\right)}\right].$$

**Figure 5:**
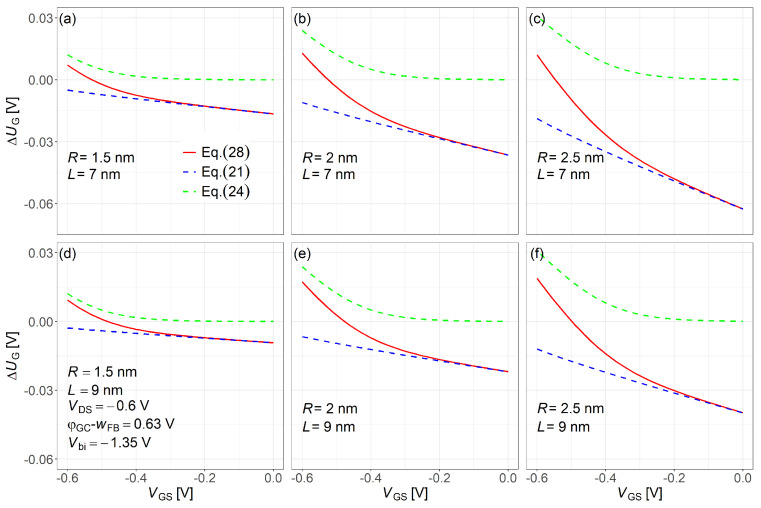
Comparison of $\Delta U_{G}^{\mathrm{all}}$ in Equation (28), $\Delta U_{G}^{\mathrm{DIBL}}\left(z_{\mathrm{EXT}}\right)$ in Equation (21) due to DIBL at $z_{\mathrm{EXT}}$ and $\Delta U_{G}^{\left(1\right)}$ in Equation (24) component without DIBL. Common simulation conditions: $V_{\mathrm{DS}}=-0.6\;V$, $V_{\mathrm{bi}}=-1.35\;V$, and $\varphi_{\mathrm{GS}}-w_{\mathrm{FB}}=0.63\;V$. Subfigures: (**a**) R=1.5nm,L=7nm; (**b**) R=2nm,L=7nm; (**c**) R=2.5nm,L=7nm; (**d**) R=1.5nm,L=9nm; (**e**) R=2nm,L=9nm; (**f**) R=2.5nm,L=9nm.

Figure 5 shows the calculated $\Delta U_{G}^{\mathrm{all}}$ (solid red line from Equation (28)), which gives the unknown parameter at the barrier minimum across all operating regions. For comparison, $\Delta U_{G}^{\mathrm{DIBL}}$ (blue dashed line from Equation (21), subthreshold region) and $\Delta U_{G}^{\left(1\right)}$ (green dashed line from Equation (24), inversion region) are also plotted. When the DIBL effect is ignored, $\Delta U_{G}^{\left(1\right)}$ becomes zero in the subthreshold region. In contrast, $\Delta U_{G}^{\mathrm{DIBL}}$ stays negative across all operating regions. As the gate length *L* decreases, the drain–channel electrostatic coupling strengthens, enhancing the DIBL effect and increasing $\Delta U_{G}^{\mathrm{DIBL}}$. Conversely, as the nanowire radius *R* decreases, quantum confinement is reinforced, which mitigates part of the DIBL-induced potential drop and leads to a smaller $\Delta U_{G}^{\mathrm{DIBL}}$ compared with that at larger *R*.

**Figure 6:**
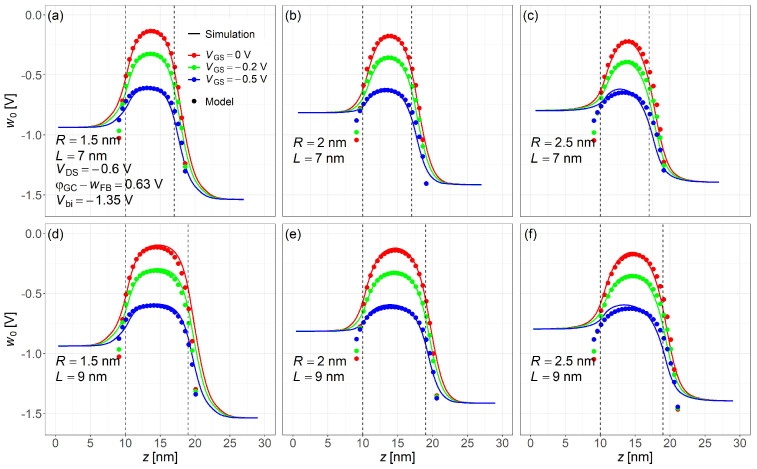
Comparison of electrostatic potential profiles along the channel center from the compact model using Equation (9) and NEGF simulation under different gate biases. Gate biases shown: $V_{\mathrm{GS}}=0$, −0.2, and −0.5V. Common simulation conditions: $V_{\mathrm{DS}}=-0.6\;V$, $V_{\mathrm{bi}}=-1.35\;V$, and $\varphi_{\mathrm{GS}}-w_{\mathrm{FB}}=0.63\;V$. Subfigures: (**a**) R=1.5nm,L=7nm; (**b**) R=2nm,L=7nm; (**c**) R=2.5nm,L=7nm; (**d**) R=1.5nm,L=9nm; (**e**) R=2nm,L=9nm; (**f**) R=2.5nm,L=9nm.

**Figure 7:**
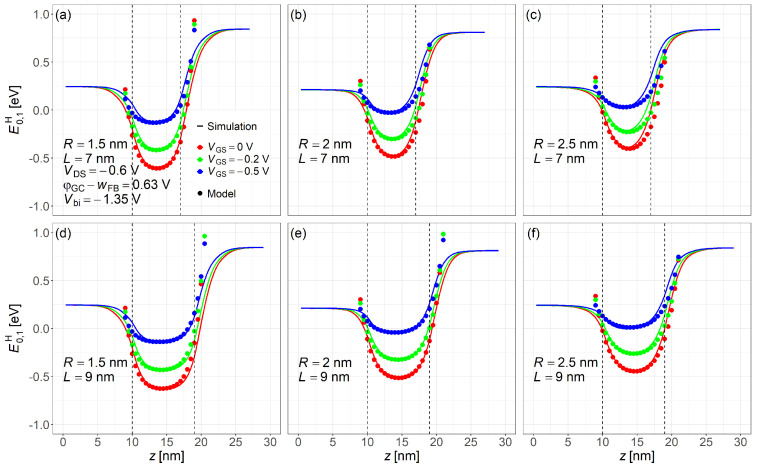
Gate bias dependence of the hole lowest subband energy level along the channel calculated by the NEGF simulation and proposed compact model. Gate biases shown: $V_{\mathrm{GS}}=0$, −0.2, and −0.5V. Common simulation conditions: $V_{\mathrm{DS}}=-0.6\;V$, $V_{\mathrm{bi}}=-1.35\;V$, and $\varphi_{\mathrm{GS}}-w_{\mathrm{FB}}=0.63\;V$. Subfigures: (**a**) R=1.5nm,L=7nm; (**b**) R=2nm,L=7nm; (**c**) R=2.5nm,L=7nm; (**d**) R=1.5nm,L=9nm; (**e**) R=2nm,L=9nm; (**f**) R=2.5nm,L=9nm.

Figure 6 compares the electrostatic potential along the channel center from the proposed model (dots, from Equation (9)) and NEGF simulations (solid lines). The vertical dashed lines indicate the source/channel and drain/channel interfaces. The red, green, and blue curves (top to bottom) represent $V_{\mathrm{GS}}$ = 0, −0.2, −0.5 V, respectively. To better match the NEGF results and include the effects of source/drain junctions, the effective channel length was extended empirically by 1 nm (source) and 2 nm (drain) for various device dimensions under all biases. These extensions account for the depletion regions and barrier lowering near the source and drain, which are not directly captured by the core model. Moreover, Figure 6 shows a diminishing radius effect: at R=1.5 nm, stronger quantum confinement raises the hole subband energy level farther above the Fermi level than for R>2 nm, leading to a clear change in carrier supply and current. When the radius increases from 2.0 to 2.5 nm, the additional confinement relief is small, so the curves differ only slightly. These observations are consistent with our NEGF results. It should be noted that the $V_{\mathrm{GS}}$ values in Figure 6 and Figure 7 are selected to demonstrate the model’s accuracy across subthreshold, linear, and saturation regions, while $I_{\mathrm{SD}}$ is calculated from the complete model (Equation (29)) rather than from these specific bias points.

Figure 7 compares the lowest subband energy profile for holes along the channel as obtained from NEGF simulations and the proposed compact model at gate biases of $V_{\mathrm{GS}}$ = 0, −0.2, −0.5 V from the bottom to top. Simulation results are well captured by the analytic compact model.

**Figure 8:**
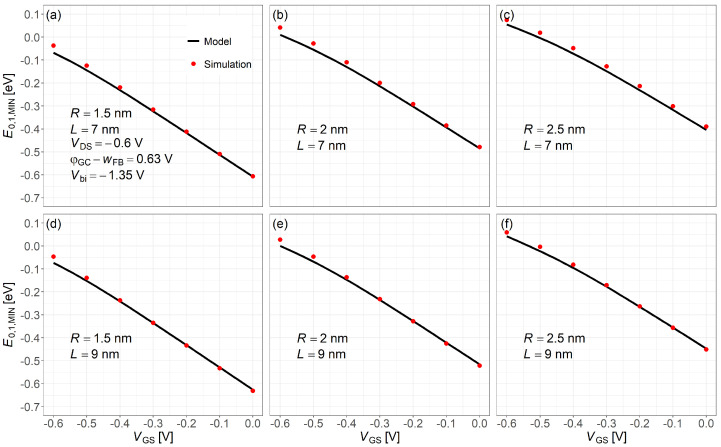
Comparison of the hole lowest subband energy level minimum obtained from NEGF simulations and the proposed compact model. Common simulation conditions: $V_{\mathrm{DS}}=-0.6\;V$, $V_{\mathrm{bi}}=-1.35\;V$, and $\varphi_{\mathrm{GS}}-w_{\mathrm{FB}}=0.63\;V$. Subfigures: (**a**) R=1.5nm,L=7nm; (**b**) R=2nm,L=7nm; (**c**) R=2.5nm,L=7nm; (**d**) R=1.5nm,L=9nm; (**e**) R=2nm,L=9nm; (**f**) R=2.5nm,L=9nm.

Figure 8 shows the lowest subband energy level for holes at the barrier minimum as a function of gate voltage, comparing NEGF simulation results (dots) with the compact model (solid lines). The results of simulation and analytic compact model agree well across the plotted range.

**Figure 9:**
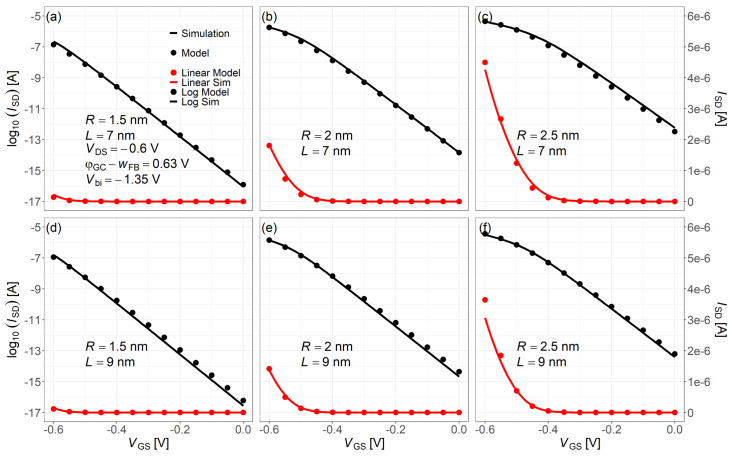
Comparison of $I_{\mathrm{SD}}-V_{\mathrm{GS}}$ between the NEGF simulation and the proposed compact model, assuming only the lowest subband is occupied. Common simulation conditions: $V_{\mathrm{DS}}=-0.6\;V$, $V_{\mathrm{bi}}=-1.35\;V$, and $\varphi_{\mathrm{GS}}-w_{\mathrm{FB}}=0.63\;V$. Subfigures: (**a**) R=1.5nm,L=7nm; (**b**) R=2nm,L=7nm; (**c**) R=2.5nm,L=7nm; (**d**) R=1.5nm,L=9nm; (**e**) R=2nm,L=9nm; (**f**) R=2.5nm,L=9nm.

Figure 9 shows the $I_{\mathrm{SD}}-V_{\mathrm{GS}}$ curves for different channel lengths and nanowire radii, calculated using Equation (29) across all operating regions. Dots represent the compact model, and solid lines are from NEGF simulations.

**Figure 10:**
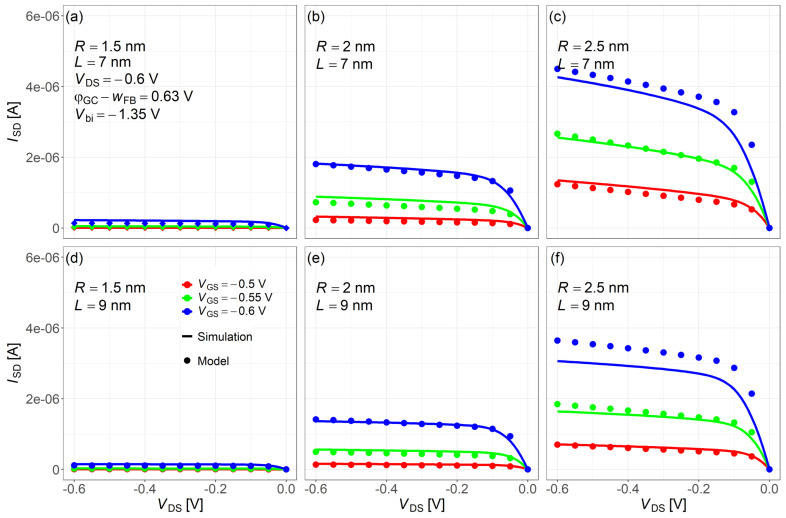
$I_{\mathrm{SD}}-V_{\mathrm{DS}}$ characteristics comparison between the NEGF simulation (solid lines) and proposed compact model (dots) for the lowest subband energy level. Common simulation conditions: $V_{\mathrm{DS}}=-0.6\;V$, $V_{\mathrm{bi}}=-1.35\;V$, and $\varphi_{\mathrm{GS}}-w_{\mathrm{FB}}=0.63\;V$. Subfigures: (**a**) R=1.5nm,L=7nm; (**b**) R=2nm,L=7nm; (**c**) R=2.5nm,L=7nm; (**d**) R=1.5nm,L=9nm; (**e**) R=2nm,L=9nm; (**f**) R=2.5nm,L=9nm.

Figure 10 shows the $I_{\mathrm{SD}}-V_{\mathrm{DS}}$ characteristics comparing NEGF simulations (solid lines) with the compact model (dots) for various channel lengths and nanowire radii. The three curves (red, green, blue from top to bottom) correspond to $V_{\mathrm{DS}}$ = −0.6, −0.55, −0.5 V, respectively. The larger deviation between panels (c) and (f) in Figure 10 is attributable to the $I_{\mathrm{SD}}-V_{\mathrm{GS}}$ mismatch around −0.5–−0.6 V in Figure 9. Because we use one global fitting parameter (no re-tuning per case), this local mismatch carries over and becomes larger in the corresponding $I_{\mathrm{SD}}-V_{\mathrm{DS}}$ curves.

**Figure 11:**
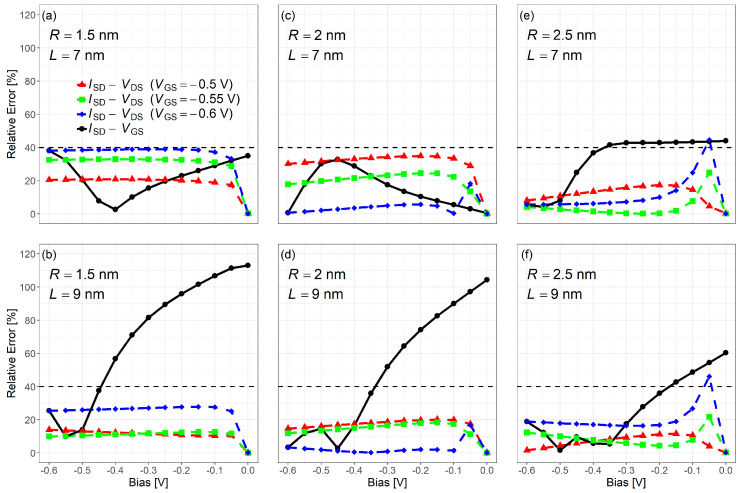
Relative errors between the proposed model and NEGF simulation for both $I_{\mathrm{SD}}-V_{\mathrm{GS}}$ and $I_{\mathrm{SD}}-V_{\mathrm{DS}}$ characteristics. Note: Deviations are reported to document the remaining error; with a single fitting parameter (no per-case re-tuning), residuals are localized rather than strictly monotonic with *R*, *L*, $V_{\mathrm{GS}}$ or $V_{\mathrm{DS}}$. Subfigures: (**a**) R=1.5nm,L=7nm; (**b**) R=2nm,L=7nm; (**c**) R=2.5nm,L=7nm; (**d**) R=1.5nm,L=9nm; (**e**) R=2nm,L=9nm; (**f**) R=2.5nm,L=9nm.

As shown in Figure 11, the relative error between the model and NEGF results for $I_{\mathrm{SD}}-V_{\mathrm{GS}}$ and $I_{\mathrm{SD}}-V_{\mathrm{DS}}$ was evaluated under different bias conditions. While the relative error exceeds 100% in the subthreshold region for some device sizes, it remains within 0–40% across most operating conditions. Since the subthreshold current is very small, such errors are practically negligible. The model demonstrate good accuracy in the inversion region for compact modeling purposes and captures key trends and bias-dependent behavior. Since a single fitting parameter is used across all cases (no per-corner re-tuning), the results show no simple monotonic dependence on *R*, *L*, $V_{\mathrm{GS}}$, $V_{\mathrm{DS}}$ and should be viewed as a wide-range one-parameter compromise.

**Figure 12:**
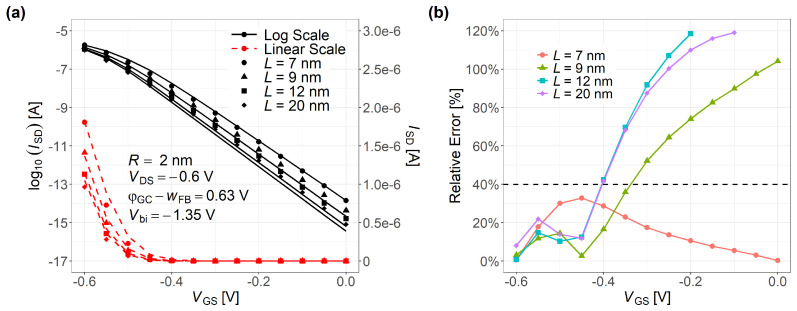
(**a**) $I_{\mathrm{SD}}$-$V_{\mathrm{GS}}$ comparison at R=2 nm with L=7,9,12,20 nm under identical $V_{\mathrm{DS}}$. (**b**) Relative error distributions for the same set.

Figure 12 demonstrates a side-by-side $I_{\mathrm{SD}}$-$V_{\mathrm{GS}}$ comparisons and error-distribution plots at R=2 nm with L=7,9,12,20 nm, respectively. After re-selecting the single fitting parameter for this wider length set, the relative error profile shifts. The errors decrease in inversion but increase in subthreshold, highlighting the trade-off and limitation of a one-parameter calibration over a wide (*R*, *L*, $V_{\mathrm{GS}}$, $V_{\mathrm{DS}}$) range. Compared with our conference paper [36], which focused on subband-energy agreement at *L* = 20 nm, the present work introduces an analytical DIBL formulation, improves accuracy, and expands validation across *R*, *L*, $V_{\mathrm{GS}}$, and $V_{\mathrm{DS}}$. These results emphasize wide-range current agreement under a single α while motivating multi-parameter and physics-refined extensions in future work.

**Figure 13:**
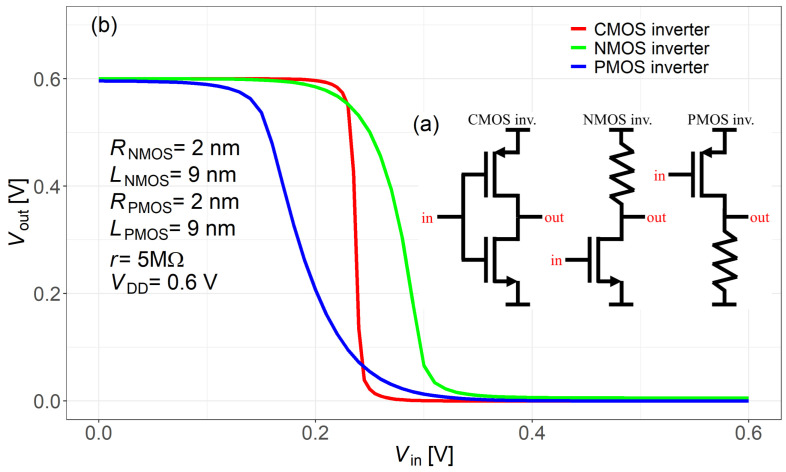
(**a**) CMOS, NMOS, and PMOS inverter schematics; (**b**) Simulated $V_{\mathrm{OUT}}-V_{\mathrm{IN}}$ characteristics from SPICE.

Figure 13a shows the circuit diagrams of the CMOS, NMOS, and PMOS inverters. Figure 13b shows the simulated output voltage ($V_{\mathrm{OUT}}$) versus input voltage ($V_{\mathrm{IN}}$) from SPICE simulations. To perform these simulations, the proposed quasi-ballistic/ballistic compact model with DIBL effect was implemented in SPICE using a Verilog-A module. Three inverters were simulated: an NMOS inverter (n-type GAA MOSFET with resistive load), a PMOS inverter (p-type GAA MOSFET with resistive load), and a CMOS inverter combining both transistors, as shown in Figure 13a. The new p-type model was used together with an existing n-type GAA model [25] to build the CMOS inverter. In Figure 13b, $V_{\mathrm{OUT}}$ versus $V_{\mathrm{IN}}$ is shown for all three inverters from SPICE DC sweep. The subscripts in *R* and *L* (NMOS or PMOS) indicate the radius and channel length of each transistor. The supply voltage is set to $V_{\mathrm{DD}}$ = 0.6 V, according to the IRDS projected range (0.55–0.65 V) [5]. The proposed Verilog-A model supports DC operation only and is applicable to static analyses such as transfer characteristics and bias points. Future work will extend it to dynamic simulations by incorporating charge-based formulations.

**Figure 14:**
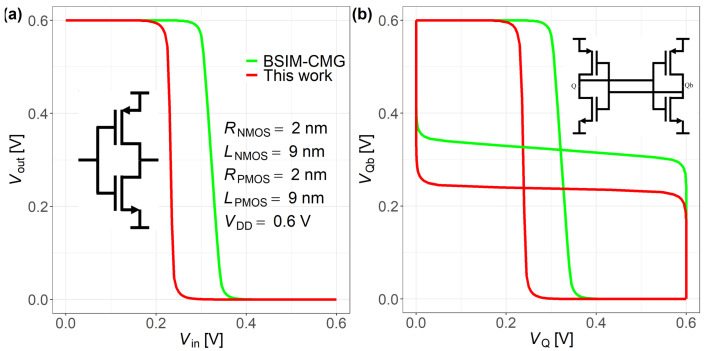
Comparisons between proposed model and BSIM-CMG model in (**a**) a CMOS inverter (**b**) a 4T SRAM simulation.

As shown in Figure 14, a CMOS inverter and a 4T SRAM butterfly SNM (Static Noise Margin) comparison between the proposed compact model and BSIM-CMG are performed under identical bias conditions. In the inverter simulations, the proposed model exhibits a slightly lower threshold voltage and a steeper near-threshold slope than BSIM-CMG. In addition, the proposed model reproduces the SNM trend across hold/read conditions. A residual offset of 90 mV appears near the near-threshold/read-disturb region. We attribute this to (i) a small threshold mismatch between the proposed compact model and BSIM-CMG and (ii) the current DC-only formulation (capacitances, leakage partition, and noise are not modeled). Moreover, the near-threshold slope of proposed model is slightly steeper than that of BSIM-CMG, which further amplifies the deviation in the read-disturb region. Outside this region, the SNM difference remains within 90 mV. Under identical SPICE settings, the runtime is comparable to BSIM-CMG (Inverter 0–0.6 V/100 steps: 0.387 s vs 0.368 s; 4T SRAM SNM: 0.392 s vs 0.410 s).

## 5. Conclusions

This work presents an analytic compact DC model for the source—drain current of p-type cylindrical gate-all-around (GAA) MOSFETs, built on the Landauer approach and explicitly including drain-induced barrier lowering (DIBL). The model fills a gap in prior studies by providing a SPICE-compatible framework for quasi-ballistic/ballistic p-type nanowire devices. It shows good agreement with non-equilibrium Green’s function (NEGF) simulations over representative channel lengths, nanowire radii, and bias ranges, and it is implemented in Verilog-A for circuit-level SPICE simulations.

In summary, the present model is exploratory and uses a single global fitting parameter, prioritizing physical clarity and efficiency over industry-level quantitative accuracy. While limited precision appears under certain bias conditions, it consistently captures the key transport trends and the primary directional behavior of p-type devices. The framework can be extended to other confinement shapes, such as nanosheet- or quantum-well-like structures, by using appropriate 2D density-of-states/subband formulas or through NEGF-guided calibration.

Future work will improve quantitative accuracy through multi-parameter calibration, the inclusion of higher-order physics such as scattering and source-to-drain tunneling, and modest smoothing and binning to preserve compactness and transparency. We will also add a charge-based AC capacitance model for small-signal and transient analyses and develop a noise-aware compact model to enable predictive nanoscale CMOS design.

## Data Availability

All simulation decks, solver options, and analysis scripts that support the findings of this study are openly available at GitHub (https://github.com/hecheng618/PGAA7nm; accessed on 16 October 2025).
